# Prostate cancer lesions in transition zone exhibit a higher propensity for pathological upgrading in radical prostatectomy

**DOI:** 10.1007/s00345-024-05294-6

**Published:** 2024-10-30

**Authors:** Xin Chen, He Wang, Chaozhong Wang, Chengbo Qian, Yuxin Lin, Yuhua Huang, Xuedong Wei, Jianquan Hou

**Affiliations:** 1 Department of Urology, The Fourth Affiliated Hospital of Soochow University (Dushu Lake Hospital Affiliated to Soochow University), No.9 Chongwen Road, Suzhou, 215006 People’s Republic of China; 2 Department of Urology, The First Affiliated Hospital of Soochow University, No. 899 Pinghai Road, Suzhou, 215006 People’s Republic of China; 3 Department of Urology, ChangShu NO.2 People’s Hospital, Suzhou, 215006 People’s Republic of China

**Keywords:** MRI, Prostate cancer, Prostate biopsy, Transition zone, Upgrading

## Abstract

**Background:**

The varying malignancy and lethality of different grades of prostate cancer (PCa) highlight the importance of accurate diagnosis. This study aims to evaluate the upgrading of transition zone (TZ) prostate cancer biopsies and identify factors to improve TZ biopsy accuracy.

**Materials and methods:**

This retrospective study included 217 patients who underwent laparoscopic radical prostatectomy after 12 + X cores transperineal transrectal ultrasound-magnetic resonance imaging (MRI)-guided targeted prostate biopsy from 2018 to 2021 in our center.

**Results:**

Patients with TZ lesions showed a higher incidence of International Society of Urological Pathology (ISUP) grade upgrading from 1 to higher grade compared to peripheral zone lesions (16.9% vs. 5.0%, *p* = 0.005). Multivariate analysis confirmed TZ lesions as an independent risk factor (OR: 4.594, 97.5% CI: 1.569–15.238, *p* = 0.008) for upgrading from 1 to higher. Additionally, the number of positive biopsy cores (OR: 0.586, 97.5% CI: 0.336–0.891, *p* = 0.029) and anterior TZ lesion location (OR: 10.797, 97.5% CI: 1.503-248.727, *p* = 0.048) were independent factors for the upgrading in TZ patients.

**Conclusions:**

This study found that PCa lesions located in the TZ, particularly the anterior TZ, have a higher risk of ISUP grade upgrading. This elevated risk arises from the insufficient distribution of biopsy cores around the TZ lesion. The findings underscore the importance of having an adequate number of biopsy cores around the lesion area to improve the accuracy of ISUP grade assessments.

**Supplementary Information:**

The online version contains supplementary material available at 10.1007/s00345-024-05294-6.

## Introduction

The incidence of prostate cancer (PCa) is increasing, with cases ranging from indolent to highly lethal behaviors [1]. The Gleason scoring system and grading criteria established by the International Society of Urological Pathology (ISUP) are valued for their prognostic significance, effectively delineating the malignancy and risk profiles of PCa [2, 3]. Low-grade PCa (ISUP grade 1) has been shown in extensive clinical trials to carry an exceedingly low risk of mortality from cancer-specific causes [4]. Research indicates that patients with ISUP grade 1 PCa have a 5-year biochemical progression-free probability of 96% [2]. In stark contrast, cancers graded 3 to 5 exhibit significantly higher malignancy [4]. The divergent malignancy and lethality associated with different grades of PCa underscore the critical importance of accurate diagnosis [5].

Despite the relentless refinements in the prostate biopsy technique, which have notably heightened the precision in diagnosing the spectrum of prostate cancer grades, the phenomenon of upgrading or downgrading the initial diagnosis upon radical prostatectomy (RP) remains prevalent [6–8]. Such diagnostic inaccuracies can lead to the risk of undertreatment when biopsies fail to detect aggressive disease. Furthermore, transrectal ultrasound-identified peripheral zone (PZ) cancers demonstrate a concordance between Gleason scores from biopsy and RP specimens in 55% of patients, whereas this agreement is observed in only 15.2% of transition zone (TZ) cancers [9]. However, advancements in multiparametric magnetic resonance imaging (Mp-MRI) technology, along with the refinement of the Prostate Imaging Reporting and Data System (PI-RADS) guidelines, have significantly informed biopsy decisions and shaped treatment strategies for PCa, enhancing the precision of lesion localization [10–13].

This study assesses the potential inaccuracy of TZ tumor biopsies and explores the factors influencing such inaccuracies. Understanding these factors may offer insights for refining biopsy techniques and enhancing the clinical accuracy of ISUP grade assessments.

## Materials and methods

### Patients

The study included 217 patients who underwent laparoscopic RP after a “12 + X” cores transperineal transrectal ultrasound-MRI-fusion biopsy between 2018 and 2021 at our center. Inclusion criteria: (1) All preoperative examinations, including blood tests, Mp-MRI, biopsy, and RP, were performed at our center; (2) PCa was confirmed by primary biopsy. Exclusion criteria: (1) PI-RADS score < 4 (MRI lesions less likely to be tumors, with positive rates of 2% for PI-RADS 1, 4% for PI-RADS 2, and 20% for PI-RADS 3 [14]); (2) Diffuse MRI lesions or unclear MRI data; (3) Prostate-specific antigen (PSA) > 100 ng/ml (due to potential inaccuracies); (4) Neoadjuvant therapy (due to changes in clinical features).

### Mp-MRI and clinical features

All patients were imaged using 3.0T MRI scanner with a standard spine array coil and an 18-channel body array coil. The required sequence was obtained as previously described [15]. A transperineal 12-core system biopsy (8 cores in the PZ, 4 cores in the TZ) and an additional X-core (2–5 cores) targeted biopsy were performed using transrectal ultrasound and MRI image fusion software [16]. All MRI parameters were measured according to the PI-RADS v2.1 guidelines [17]. Pathological criteria followed the Society of Urogenital Pathology and ISUP guidelines for prostate cancer pathology [18]. All cases were reviewed by two experienced radiologists or pathologists, with additional experts consulted in case of disagreement.

For analytical purposes, ISUP grade upgrading was categorized based on risk stratification guidelines from the National Comprehensive Cancer Network (NCCN) and the European Association of Urology (EAU) into three classes: any grade upgrade, upgrading from grade 1 to higher, and upgrading from grades 2 or 3 to grades 4 or 5. These upgrades influence tumor risk stratification and, consequently, treatment strategies [19, 20].

### Statistical analyses

Continuous variables were analyzed using t-tests for normally distributed data and nonparametric tests for non-normally distributed data. Categorical variables were analyzed using the Chi-square or Fisher’s exact test. Data analysis was performed using GraphPad Prism (version 8.0.2) and R (version 4.1.2). A significance level of *P* < 0.05 was considered statistically significant.

## Results

### Descriptive analysis of patients

The clinical characteristics of all patients are shown in Table 1.

**Table 1:** Characteristics of the study cohort

Variable	Result	
Study patients, n (%)	217	
Median age, yr (IQR)	69	(65, 75)
Median initial tPSA, ng/ml (IQR)	17	(7, 22)
Median numbers of positive cores (systematic), n (IQR)	4	(2, 6)
Biopsy ISUP grade, n (%)		
1	30	(14)
2	79	(37)
3	61	(28)
4	29	(13)
5	18	(8)
Radical prostatectomy ISUP grade, n (%)		
1	10	(5)
2	62	(28)
3	83	(38)
4	28	(13)
5	34	(16)
**Mp-MRI report characteristics**		
PI-RADS score, n (%)		
4	92	(42)
5	125	(58)
Mapping lesions, n (%)		
PZ	121	(56)
TZ	83	(38)
PZ + TZ	13	(6)
Median Largest dimension of lesion, mm (IQR)	15	(11, 19)
Median lesion volume, ml (IQR)	0.8	(0.3, 1.9)
Median prostate volume, ml (IQR)	32	(23, 42)
Median TZ volume, ml (IQR)	13	(8, 20)
Median PZ volume, ml (IQR)	18	(13, 23)
Median lesion volume ratio, % (IQR)	3	(1, 6)
Median TZ volume ratio, % (IQR)	41	(33, 53)
Median PZ volume ratio, % (IQR)	59	(47, 67)
Median PSA density, ng/ml^2 (IQR)	0.4	(0.2, 0.7)

IQR, interquartile; PSA, Prostate-specific antigen; ISUP, International Society of Urological Pathology; PI-RADS, Prostate Imaging Reporting and Data System; TZ or PZ patients, patients’ index lesions of MRI in the TZ or PZ; PZ + TZ patients, patients’ multiple lesions of MRI mapping lesions in the PZ and TZ

### Patients with TZ lesions demonstrate a higher incidence of upgrading from grade 1 to higher and 2, 3 to 4, 5 compared to PZ lesions

**Fig. 1:**
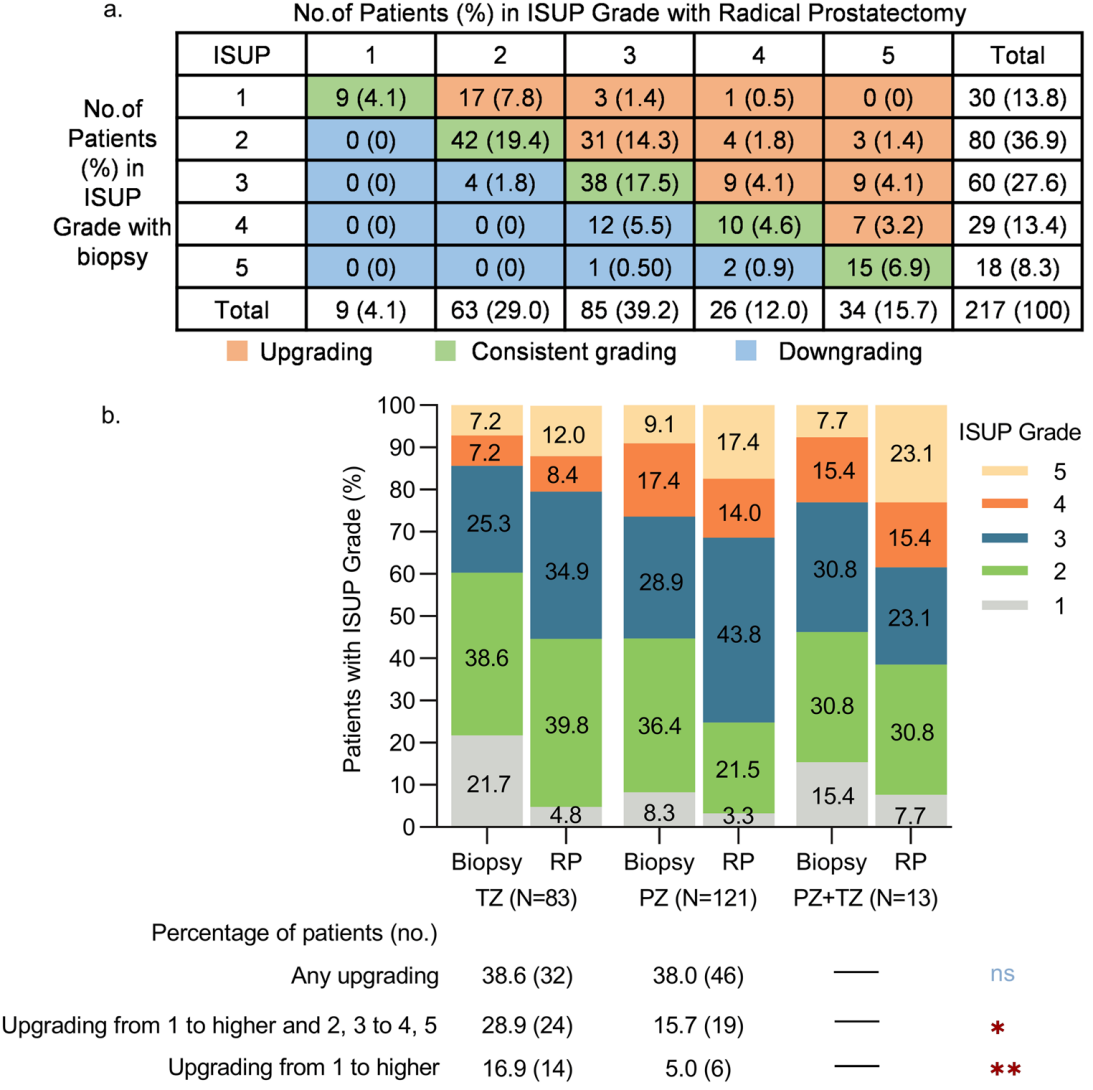
The ISUP grade upgradation of enrolled cases. (**a**) An overview of the upgrading, downgrading, and consistent grading of enrolled cases. (**b**) The upgrading among the PZ, TZ, and PZ + TZ subgroups. Patients with TZ lesions experienced a higher rate of upgrading from 1 to higher and 2, 3 to 4, 5 compared to PZ lesions (Details in Online Resource 2). (*: *p* < 0.05, **: *p* < 0.01)

Among the enrolled patients, 84 cases (38.7%) experienced an upgrading in the ISUP grade upon RP pathology, while 19 cases (8.8%) showed a downgrading, and 114 cases (52.5%) had consistent grading. The rate of upgrading was significantly higher than that of downgrading (38.7% vs. 8.8%, *p* < 0.001). Furthermore, patients with ISUP grades 1 experienced a higher rate of upgradation compared to those with grades 2, 3 and 4 (70.0% vs. 47.5% vs. 30.0% vs. 24.1%, respectively, *p* < 0.05). Similarly, ISUP grade 2 patients showed a higher upgradation rate than those with grades 3 and 4 (47.5% vs. 30.0% vs. 24.1%, respectively, *p* < 0.05). (Fig. 1, details in Online Resource 1)

Subgroup analysis of patients with lesions located in the TZ, PZ, and TZ + PZ revealed that patients with TZ lesions experienced a higher rate of upgrading from 1 to higher and 2, 3 to 4, 5 (28.9% vs. 15.7%, *p* = 0.021) and a higher rate of upgrading from 1 to higher (16.9% vs. 5.0%, *p* = 0.005) compared to PZ lesions (Fig. 1, details in Online Resource 2).

Univariate logistic regression analysis identified TZ lesions as a risk factor for upgrading from 1 to higher and from 2, 3 to 4, 5 in enrolled patients (OR: 2.698, 97.5% CI: 1.394–5.315, *p* = 0.004). In multivariate analysis, TZ lesions were also an independent risk factor (OR: 2.406, 97.5% CI: 1.182–4.980, *p* = 0.016). Additionally, univariate analysis identified TZ lesions as a risk factor for upgrading from 1 to higher (OR: 4.601, 97.5% CI: 1.783–13.398, *p* = 0.003), and multivariate analysis confirmed them as an independent risk factor (OR: 4.594, 97.5% CI: 1.569–15.238, *p* = 0.008). (Online Resource 3–4).

### Upgrading rates of TZ tumors vary by biopsy methods and TZ subregion

**Fig. 2:**
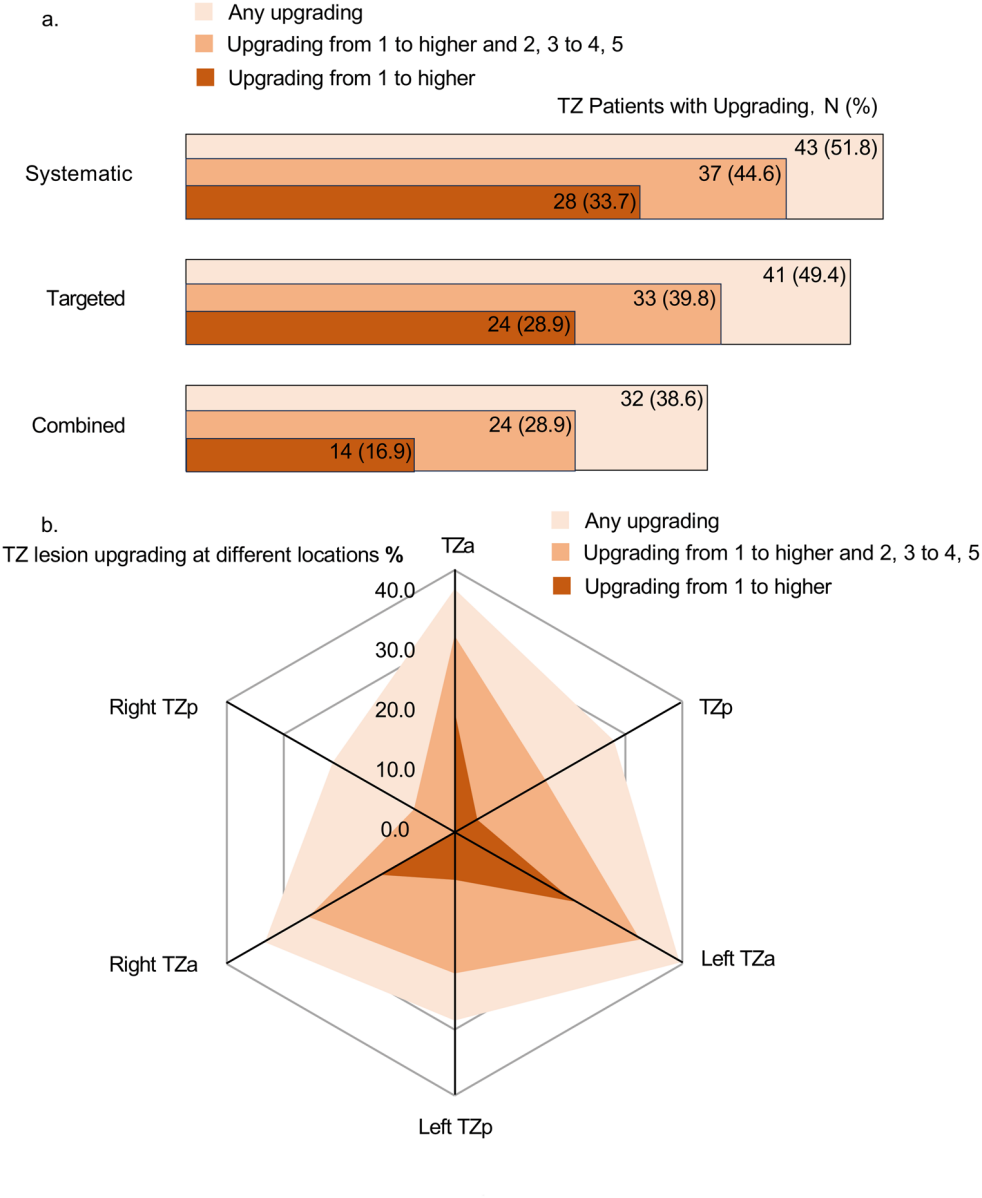
The upgrading rates of different biopsy techniques and various sub-regions in the TZ tumor. (**a**) Combined biopsies tend to have lower rates of upgrading compared to systematic biopsies alone and targeted biopsies alone (details in Online Resource 5). (**b**) There was a trend towards higher rates of upgrading from 1 to higher and 2, 3 to 4, 5 in the TZa compared to the TZp (details in Online Resource 6)

In a detailed analysis of patients with TZ lesions, combined systematic and targeted biopsies tend to have lower rates of upgrading compared to systematic biopsies alone and targeted biopsies alone (Combined biopsies vs. Systematic biopsies and Targeted biopsies: [Upgrading] 38.6% vs. 51.8% and 49.4%, *p* = 0.08, *p* = 0.15; [Upgrading from 1 to higher and 2, 3 to 4, 5] 28.9% vs. 44.6% and 39.8%, *p* = 0.03, *p* = 0.14; [Upgrading from 1 to higher] 16.9% vs. 33.7% and 28.9%, *p* = 0.01, *p* = 0.06) (Fig. 2, details in Online Resource 5).

Comparing the upgrading rates across different zones of the TZ, no significant difference was observed in the upgrading rates between the TZ anterior (TZa) and TZ posterior (TZp) (31.7% vs. 28.0%, *p* = 0.410). However, there was a trend towards higher rates of upgrading from 1 to higher and 2, 3 to 4, 5 in the TZa compared to the TZp (Upgrading from 1 to higher and 2, 3 to 4, 5: 30.0% vs. 12.5%, *p* = 0.109; Upgrading from 1 to higher: 18.6% vs. 4.0%, *p* = 0.104) (Fig. 2, details in Online Resource 6).

### Factors associated with upgrading in TZ tumors

**Fig. 3:**
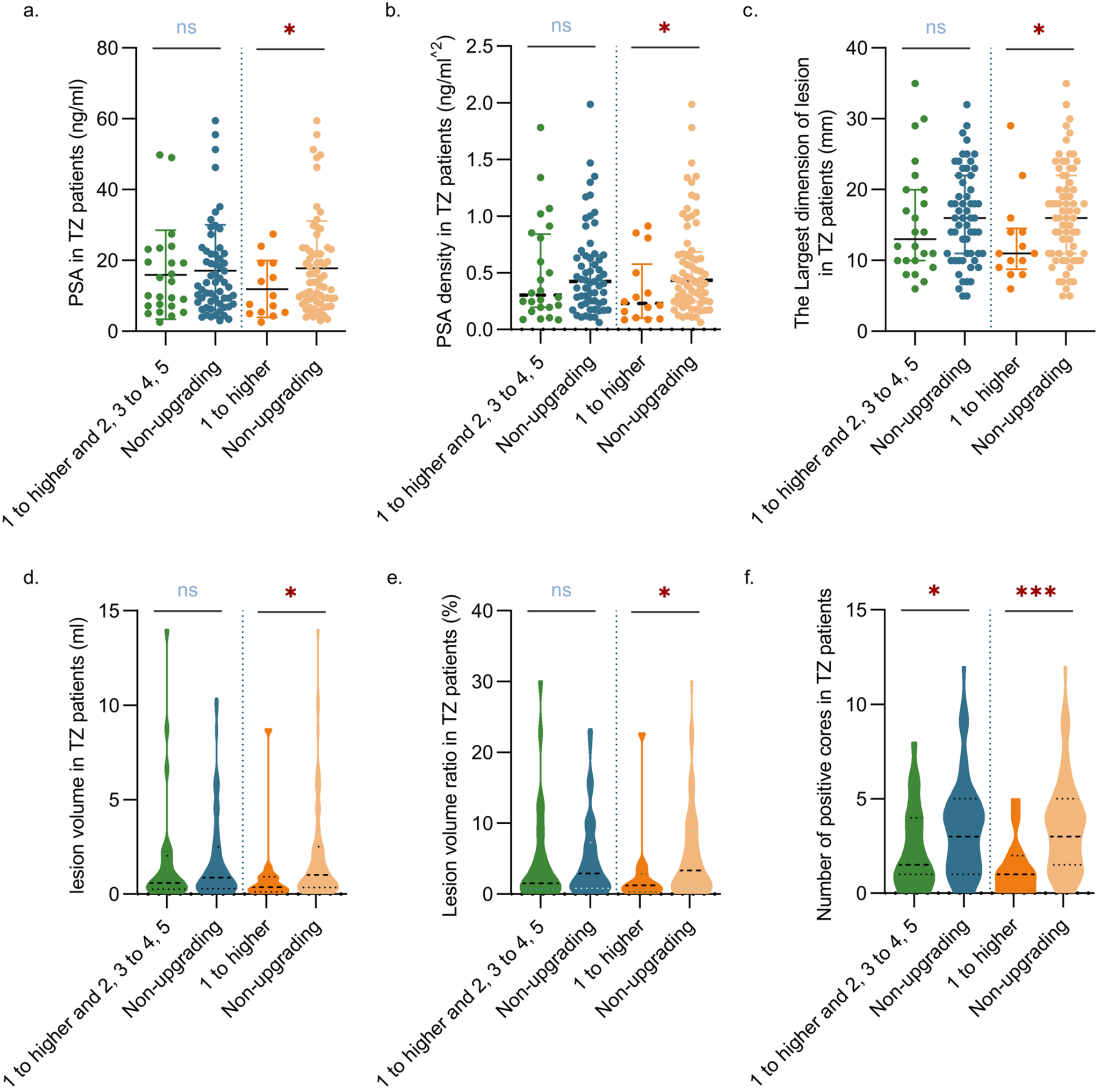
Factors associated with important and significant upgrading in TZ tumors. (**a**) Lower PSA levels were associated with upgrading from 1 to higher. (**b**) Lower PSA density was linked to upgrading from 1 to higher. (**c**) A smaller largest lesion diameter was related to upgrading from 1 to higher. (**d**) Lower lesion volume was associated with upgrading from 1 to higher. (**e**) A smaller lesion volume ratio indicated a likelihood of upgrading from 1 to higher. (**f**) Fewer positive biopsy cores were associated with upgrading from 1 to higher and 2, 3 to 4, 5. (*: *p* < 0.05, ***: *p* < 0.001)

A comparative analysis of clinical profiles was conducted among patients with TZ tumors to identify factors associated with ISUP grade upgrading. In patients with upgrading from 1 to higher and 2, 3 to 4, 5, there were fewer positive cores compared to those without important upgrading (2 vs. 3, *p* = 0.037). However, there were no statistically significant differences in terms of PSA levels, PSA density, largest lesion diameter, lesion volume, lesion volume ratio, prostate volume, TZ volume, TZ volume ratio, age, and the free-to-total PSA ratio (f/t PSA) (*p* > 0.05) between patients with and without upgrading from 1 to higher and 2, 3 to 4, 5 (Fig. 3 and Online Resource 7).

Patients with upgrading from 1 to higher had lower PSA (11.91 vs. 17.79 ng/ml, *p* = 0.036), PSA density (0.23 vs. 0.43 ng/ml^2, *p* = 0.027), largest lesion diameter (11 vs. 16 mm, *p* = 0.040), lesion volume (0.37 vs. 1.02 ml, *p* = 0.040), lesion volume ratio (1.26% vs. 3.36%, *p* = 0.027), and fewer positive biopsy cores (1 vs. 3, *p* = 0.001) compared to those without significant upgrading. However, no statistically significant differences were found in prostate volume, TZ volume, TZ volume ratio, age, and f/t PSA (*p* > 0.05) between patients with and without upgrading from 1 to higher **(**Fig. 3and Online Resource 7).

Multivariate logistic regression analysis of these indicators revealed that the number of positive biopsy cores (OR: 0.586, 97.5% CI: 0.336–0.891, *P* = 0.029) and lesion location in the TZa (OR: 10.797, 97.5% CI: 1.503-248.727, *p* = 0.048) were independent factors for upgrading from 1 to higher in TZ patients (Online Resource 8).

## Discussion

Discrepancies in ISUP grade between RP specimens and biopsy specimens can lead to under-treatment or over-treatment of patients [8, 21]. Research by Porcaro, A.B. et al. demonstrated that the upgrading was independent risk factors for PCa progression (hazard ratio [HR]: 5.95, *p* = 0.002) [21]. In this study, we found that more patients with PI-RADS > 3 experienced an upgrade in the pathological ISUP grade of their RP specimens compared to a downgrade. Particularly, the rates of upgrading from grade 1 to higher and from grades 2, 3 to 4, 5 were significantly higher in TZ patients compared to PZ patients. Investigation into the causes of upgrading in TZ patients revealed that fewer positive core biopsies and lesion location in the TZa were significant factors. This suggests that PCa lesions in the TZ, especially the TZa, have a higher risk of ISUP grade upgrading due to insufficient biopsy core distribution. This finding emphasizes the need for a more comprehensive distribution of biopsy cores around TZ lesions to obtain sufficient samples, offering valuable insights for improving biopsy strategies for TZ lesions.

The consistency rate of grading in this study aligns with previous studies, but the upgrade rate was slightly higher (38.7% vs. 31.3-33.8%) and the downgrade rate was slightly lower (8.8% vs. 14.3%) than previously reported [8, 21, 22]. This discrepancy may be attributed to our inclusion criteria, which only considered patients with a PI-RADS score > 3. Our findings also indicate that for patients with a PI-RADS score > 3, ISUP grade upgrading is more prevalent than downgrading.

Different starting points and degrees of upgrades after RP affect the corresponding risk levels and their implications. Our study found higher rates of upgrading from grade 1 to higher and from grades 2, 3 to grades 4, 5 in TZ tumors compared to PZ tumors. This suggests that TZ tumors are more likely to impact tumor risk stratification through upgrading, influencing treatment strategy decisions.

The biopsy method significantly affects the accuracy of biopsy ISUP grading. Combined biopsy resulted in fewer upgraded patients compared to both targeted biopsy alone and systematic biopsy. These findings are consistent with recent large prospective randomized clinical trials, further emphasizing the importance of combined biopsy [7, 23].

Additionally, we found that tumors located in the TZa were more prone to upgrading, and this location was an independent risk factor for upgrading. Anterior prostate cancer has been shown to have a significantly higher incidence of tumors, with only 6.76% being low-risk tumors not requiring radical treatment [24].

Moreover, it has been reported that upgrading was associated with the number of positive biopsy cores and fewer than three positive cores being a risk factor for upgrading [25]. Our study indicates that biopsy of TZ tumors may not capture the most significant pathological features due to insufficient biopsy core distribution in some cases. This finding aligns with our observation that patients with significant upgrading of TZ tumors had smaller lesion diameters, lesion volumes, and lesion volume ratios.

Overall, for TZ tumors, increasing the distribution of biopsy cores around the TZ lesion may be effective strategies to reduce ISUP grade upgrading. This result supports the recent EAU guidelines advocating for increased biopsies in lesion areas [20].

The present study has a few limitations. First, to ensure the correspondence between MRI lesions and real lesions, we excluded some patients with PI-RADS 3 and normal prostate MRI results, potentially omitting some PCa patients. Secondly, as a high-volume referral medical center, the disease patterns observed at our institution may not accurately reflect those in the community. Finally, this is a single-center retrospective study, and the findings require validation through multicenter and prospective studies.

## Conclusion

This study found that PCa lesions located in the TZ, particularly the anterior TZ, have a higher risk of ISUP grade upgrading. This elevated risk arises from the insufficient distribution of biopsy cores around the TZ lesion. The findings underscore the importance of having an adequate number of biopsy cores around the lesion area to improve the accuracy of ISUP grade assessments.

## Electronic supplementary material

Below is the link to the electronic supplementary material.

Supplementary Material 1

## Data Availability

No datasets were generated or analysed during the current study.
