# Supplementary material for: Prostate cancer lesions in transition zone exhibit a higher propensity for pathological upgrading in radical prostatectomy
Source: World J Urol. 2024 Oct 30;42(1):608. doi: 10.1007/s00345-024-05294-6 (PMC11525276; doi:10.1007/s00345-024-05294-6)
Supplement: Supplementary file 1 — Supplementary Material 1 [file 345_2024_5294_MOESM1_ESM.pdf]

Supplementary Information

Title Page:

Title: Prostate cancer lesions in transition zone exhibit a higher propensity for pathological upgrading in radical prostatectomy

Authors' names: Xin Chen[1, 2, *], He Wang[2, *], Chaozhong Wang[3], Chengbo Qian[2], Yuxin Lin[2], Yuhua Huang[2, #], Xuedong Wei[2, #], Jianquan Hou[1, 2, #]

Authors' affiliations:

[1] Department of Urology, The Fourth Affiliated Hospital of Soochow University, Dushu Lake Hospital Affiliated to Soochow University, Suzhou, 215006, People's Republic of China;

[2] Department of Urology, The First Affiliated Hospital of Soochow University, Suzhou, 215006, People's Republic of China;

[3] Department of Urology, ChangShu NO.2 People's Hospital, Suzhou, 215006, People's Republic of China;

* These authors contributed equally to this work.

# These authors are co-authors of communication.

Corresponding author:

Jianquan Hou, Department of Urology, The Fourth Affiliated Hospital of Soochow University (Dushu Lake Hospital Affiliated to Soochow University), No. No.9 Chongwen Road, Suzhou, 215006, People's Republic of China, Tel/Fax +86 512-67972184, Email xf192@163.com

Xuedong Wei, Department of Urology, The First Affiliated Hospital of Soochow University, No. 899 Pinghai Road, Suzhou, 215006, People's Republic of China, Tel/Fax +86 512-67972184, Email wxd0422@163.com / wxd0422@suda.edu.cn

Yuhua Huang, Department of Urology, The First Affiliated Hospital of Soochow University, No. 899 Pinghai Road, Suzhou, 215006, People's Republic of China, Tel/Fax +86 512-67972184, Email sdfyyhyh@163.com

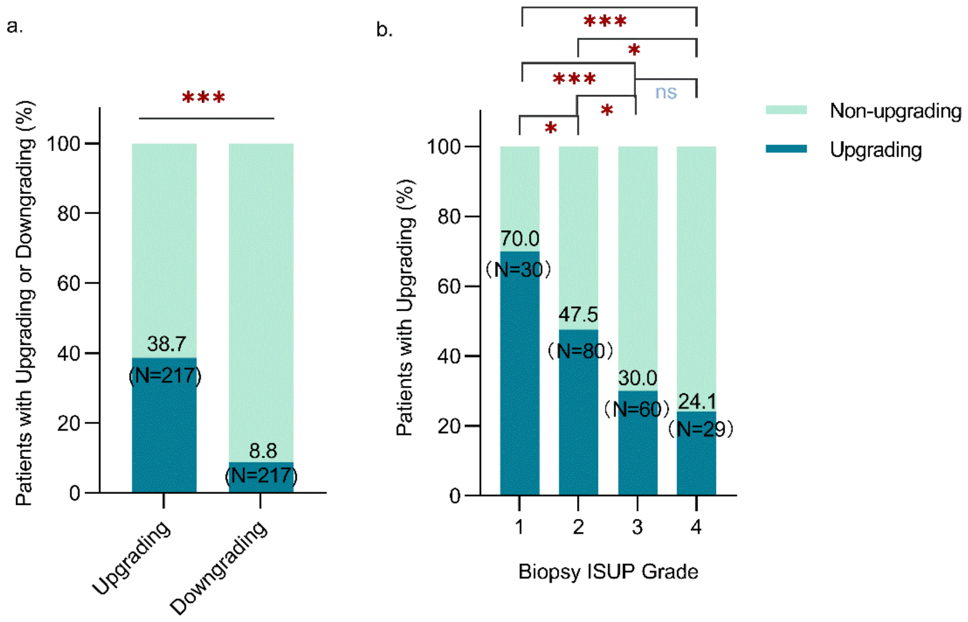

Online Resource 1. The ISUP grade upgradation of enrolled cases. (a) The rate of upgrading was significantly higher than that of downgrading (38.7% vs. 8.8%, p<0.001). (b) Patients with ISUP grades 1 experienced a higher rate of upgradation compared to those with grades2, 3 and 4 (70.0% vs. 47.5% vs. 30.0% vs. 24.1%, respectively, p<0.05). ISUP grade 2 patients showed a higher upgradation rate than those with grades 3 and 4 (47.5% vs. 30.0% vs. 24.1%, respectively, p<0.05). (ISUP, International Society of Urological Pathology; *: p<0.05, ***: p<0.001).

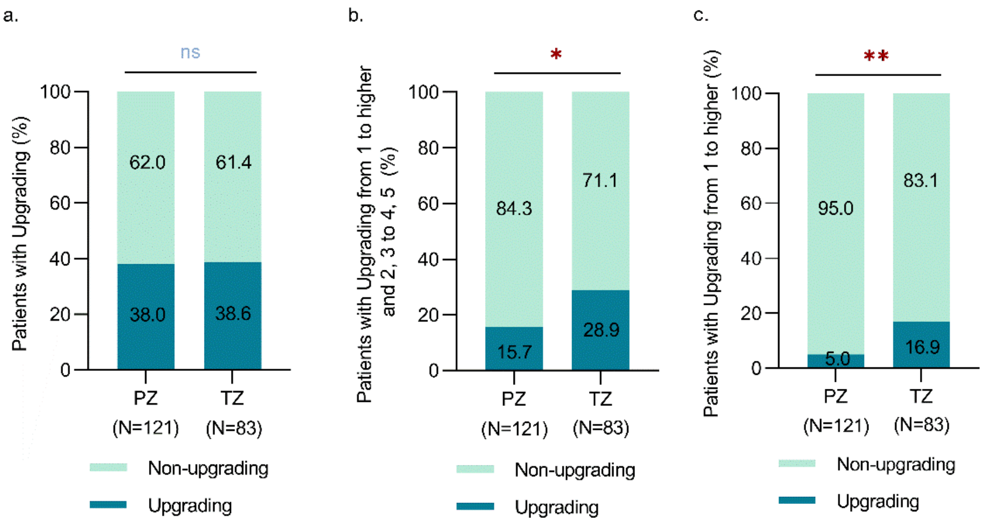

Online Resource 2. Upgrading rates in patients with TZ vs. PZ lesions. (a) No difference in upgrading rates between TZ and PZ lesions (62.0% vs. 61.4%, p=0.983). (b) Patients with TZ lesions experienced a higher rate of upgrading from 1 to higher and 2, 3 to 4, 5 compared to PZ lesions (28.9% vs. 15.7%, P=0.021). (c) Patients with TZ lesions experienced a higher rate of upgrading from 1 to higher compared to PZ lesions (16.9% vs. 5.0%, P=0.005). (TZ, Transitional Zone; PZ, Peripheral Zone; *: p<0.05, **: p<0.01)

Online Resource 3. Univariate and multivariate logistic regression analysis of factors associated with upgrading from 1 to higher and 2, 3 to 4, 5 in enrolled patients

factor	Single factor logistic regression analysis					Multivariate logistic regression analysis				
	OR	2.5% OR	97.5% OR	P		OR	2.5% OR	97.5% OR	P	
Age	0.970	0.923	1.019	0.227		0.960	0.906	1.016	0.165	
tPSA	1.001	0.976	1.024	0.916		1.048	0.965	1.143	0.276	
f/tPSA	17.194	0.273	953.751	0.162		8.869	0.050	934.875	0.374	
largest dimension of lesion	1.009	0.958	1.060	0.738		1.004	0.910	1.108	0.933	
lesion volume	1.034	0.899	1.169	0.612		0.764	0.433	1.274	0.320	
Prostate volume	1.011	0.996	1.027	0.144		1.008	0.922	1.099	0.857	
TZ volume	1.013	0.992	1.034	0.199		0.985	0.870	1.112	0.810	
lesion volume ratio	1.004	0.952	1.051	0.870		1.108	0.912	1.341	0.286	
TZ volume ratio	1.011	0.987	1.035	0.375		1.011	0.958	1.070	0.680	
PSA density	0.690	0.289	1.425	0.360		0.224	0.012	2.792	0.276	
Number of positive cores	0.910	0.796	1.033	0.155		0.964	0.823	1.122	0.645	
lesion in TZ	2.698	1.394	5.315	0.004	**	2.406	1.182	4.980	0.016	*

OR, Odds Ratio; CI, Confidence Interval; tPSA, total Prostate-Specific Antigen; f/tPSA, free PSA /total PSA; TZ, Transitional Zone; PZ, Peripheral Zone; *: $p<0.05$, **: $p<0.01$.

Online Resource 4. Univariate and multivariate logistic regression analysis of factors associated with upgrading from 1 to higher in enrolled patients

factor	Single factor logistic regression analysis					Multivariate logistic regression analysis				
	OR	2.5% OR	97.5% OR	P		OR	2.5% OR	97.5% OR	P	
Age	0.957	0.895	1.024	0.200		0.968	0.893	1.050	0.433	
tPSA	0.952	0.893	0.998	0.084		0.995	0.848	1.162	0.955	
f/tPSA	251.380	2.045	28014.300	0.019	*	83.534	0.076	36048.169	0.170	
Largest dimension of lesion	0.919	0.837	0.996	0.054		0.933	0.786	1.103	0.416	
lesion volume	0.799	0.517	1.042	0.198		0.669	0.195	1.674	0.452	
Prostate volume	1.020	1.001	1.039	0.028	*	1.058	0.934	1.197	0.371	
TZ volume	1.025	1.000	1.049	0.037	*	0.945	0.804	1.110	0.489	
lesion volume ratio	0.898	0.762	1.000	0.118		1.176	0.800	1.684	0.376	
TZ volume ratio	1.022	0.989	1.055	0.194		1.019	0.944	1.102	0.631	
PSA density	0.097	0.010	0.523	0.019	*	0.378	0.002	34.586	0.703	
Number of positive cores	0.773	0.616	0.942	0.016	*	0.985	0.761	1.249	0.904	
lesion in TZ	4.601	1.783	13.398	0.003	**	4.594	1.569	15.238	0.008	**

OR, Odds Ratio; CI, Confidence Interval; tPSA, total Prostate-Specific Antigen; f/tPSA, free PSA /total PSA; TZ, Transitional Zone; PZ, Peripheral Zone; *: p<0.05, **: p<0.01.

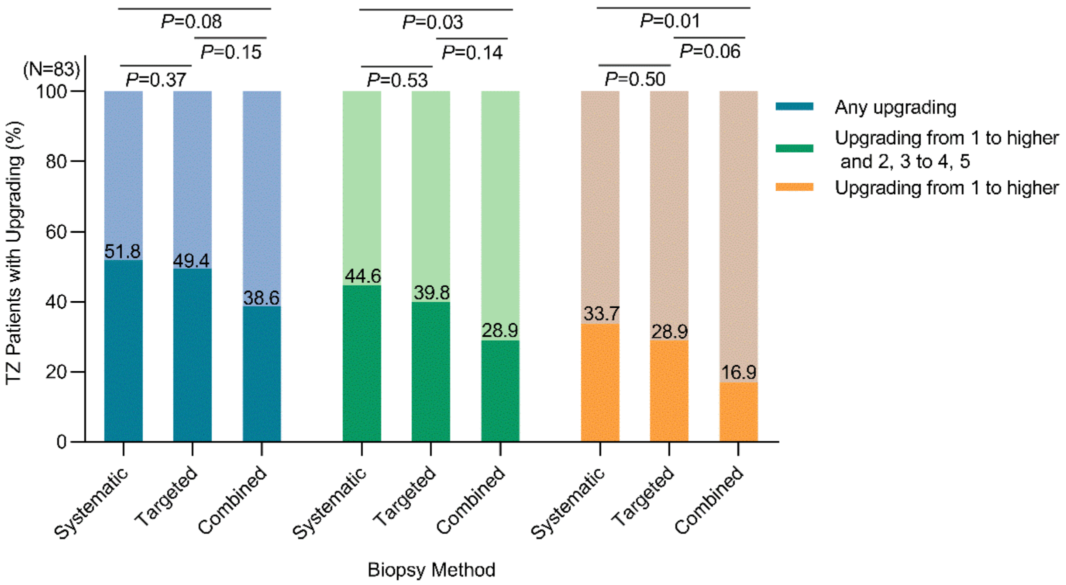

Online Resource 5. Upgrading rates in TZ patients with combined systematic and targeted biopsies vs. systematic and targeted biopsies alone. Combined biopsies tend to have lower rates of: Upgrading (38.6% vs. 51.8% and 49.4%, p=0.08, p=0.15); Upgrading from 1 to higher and 2, 3 to 4, 5 (28.9% vs. 44.6% and 39.8%, p=0.03, p=0.14); Upgrading from 1 to higher (16.9% vs. 33.7% and 28.9%, p=0.01, p=0.06). (TZ, Transitional Zone; *: p<0.05, **: p<0.01)

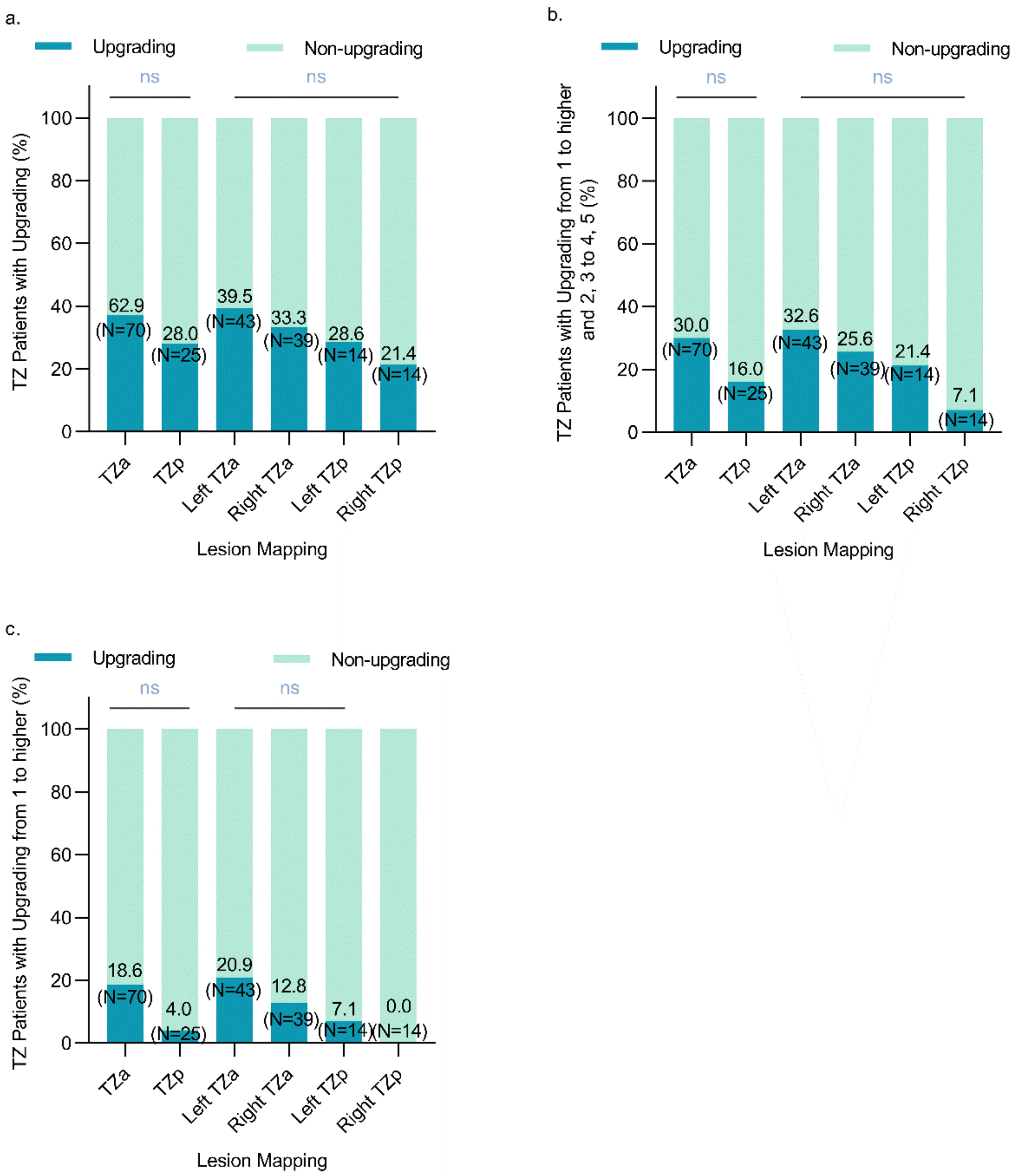

Online Resource 6. Upgrading rates in different zones of the TZ. (a) No significant difference in upgrading rates between the TZ anterior (TZa) and TZ posterior (TZp) (31.7% vs. 28.0%, p=0.410). (b) Trend towards higher rate of upgrading from 1 to higher and 2, 3 to 4, 5 in the TZa compared to the TZp (30.0% vs. 12.5%, p=0.109). (c) Trend towards higher rate of upgrading from 1 to higher in the TZa compared to the TZp (18.6% vs. 4.0%, p=0.104). (TZ, Transitional Zone; TZa, TZ Anterior; TZp, TZ Posterior)

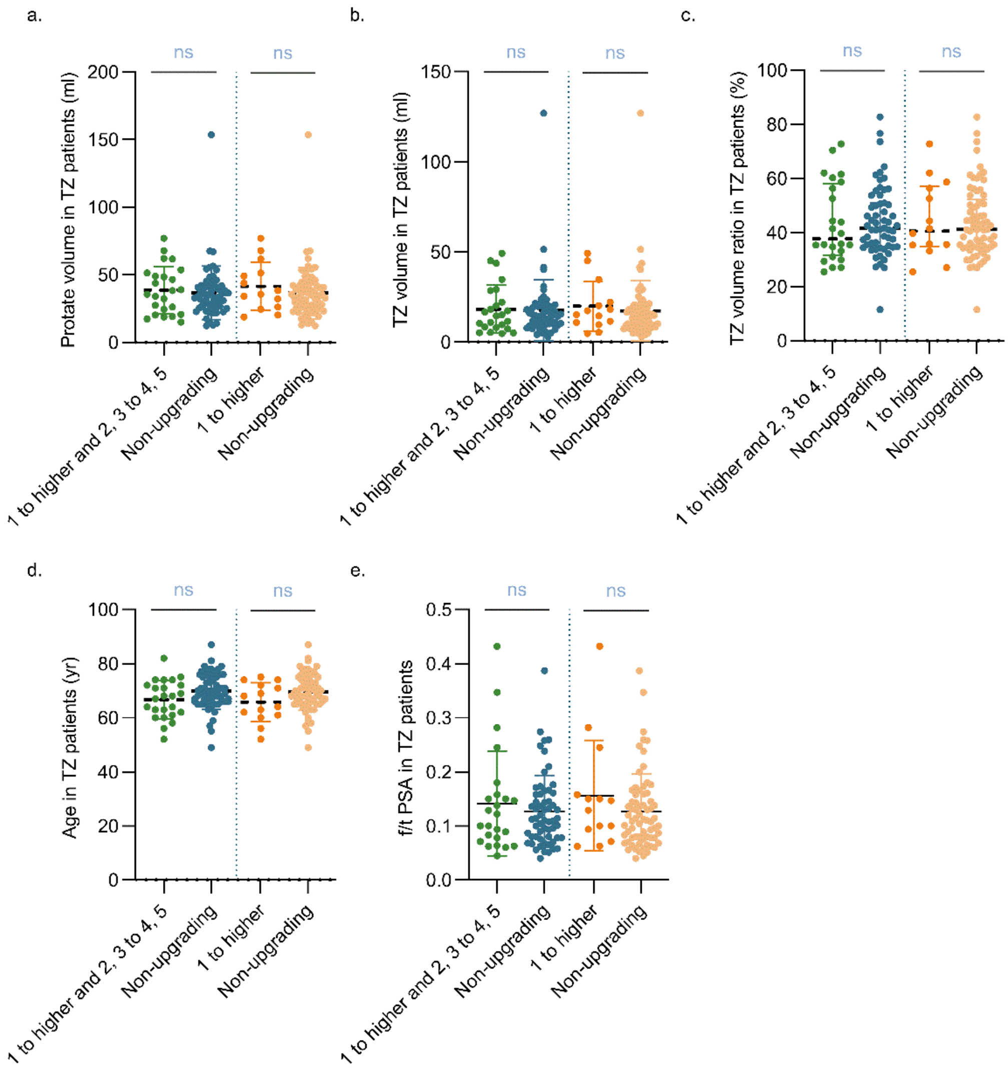

Online Resource 7. Factors associated with upgrading in TZ tumors. There were no statistically significant differences in terms of prostate volume (37 vs. 34 ml, P=0.544), TZ volume (14.83 vs. 14.48 ml, P=0.856), TZ volume ratio (37.69 vs. 41.67, P=0.540), age (67 vs. 70 years, P=0.056), and the free-to-total PSA ratio (f/t PSA) (0.11 vs. 0.11, P=0.934) between patients with and without upgrading from 1 to higher and 2, 3 to 4, 5. No statistically significant differences were found in prostate volume (37 vs. 34 ml, P=0.300), TZ volume (16.28 vs. 14.41 ml, P=0.364), TZ volume ratio (40.54 vs. 41.14, P=0.930), age (66 vs. 70 years, P=0.060), and f/t PSA (0.14 vs. 0.11, P=0.342) between patients with and without upgrading from 1 to higher.

Online Resource 8. Univariate and multivariate logistic regression analysis of factors associated with upgrading from 1 to higher in TZ patients

factor	Single factor logistic regression analysis					Multivariate logistic regression analysis				
	**OR**	**2.5% OR**	**97.5% OR**	**P**		**OR**	**2.5% OR**	**97.5% OR**	**P**	
Age	0.922	0.842	1.004	0.066		0.927	0.823	1.039	0.192	
tPSA	0.948	0.876	1.005	0.126		1.013	0.765	1.344	0.927	
f/tPSA	84.544	0.071	7.350E+04	0.195		241.959	0.004	2.650E+07	0.330	
largest dimension of lesion	0.910	0.814	1.000	0.069		0.886	0.686	1.123	0.318	
lesion volume	0.802	0.499	1.062	0.232		1.108	0.217	3.869	0.878	
Prostate volume	1.012	0.983	1.041	0.373		1.107	0.909	1.391	0.326	
TZ volume	1.008	0.970	1.040	0.593		0.858	0.587	1.112	0.285	
lesion volume ratio	0.901	0.751	1.015	0.161		0.996	0.543	1.862	0.866	
TZ volume ratio	1.002	0.958	1.045	0.924		1.050	0.929	1.240	0.988	
PSA density	0.164	0.013	1.024	0.097		2.103	2.397E-04	1.467E+04	0.460	
Number of positive cores	0.529	0.321	0.774	0.004	**	0.586	0.336	0.891	0.029	*
lesion in TZa	6.933	1.262	129.720	0.070		10.797	1.503	248.727	0.048	*

OR, Odds Ratio; CI, Confidence Interval; tPSA, total Prostate-Specific Antigen; f/tPSA, free PSA /total PSA; TZ, Transitional Zone; PZ, Peripheral Zone; TZa, TZ anterior; *: $p<0.05$, **: $p<0.01$.
